# Prevalence and Risk Factors of Sensory Symptoms in Diabetes Patients in Taiwan

**DOI:** 10.3389/fendo.2020.580426

**Published:** 2021-01-08

**Authors:** Chin-Hsiao Tseng, Choon-Khim Chong, Jau-Jiuan Sheu

**Affiliations:** ^1^ Department of Internal Medicine, National Taiwan University College of Medicine, Taipei, Taiwan; ^2^ Department of Internal Medicine, National Taiwan University Hospital, Taipei, Taiwan; ^3^ Division of Environmental Health and Occupational Medicine of the National Health Research Institutes, Zhunan, Taiwan; ^4^ Chong’s Physical Medicine and Rehabilitation Center, Taipei, Taiwan; ^5^ Department of Neurology, Taipei Medical University Hospital, Taipei, Taiwan

**Keywords:** diabetes mellitus, sensory symptoms, epidemiology, risk factors, Taiwan

## Abstract

**Background:**

Diabetic sensory neuropathy has rarely been studied in the Asian populations. This study investigated the prevalence and risk factors of sensory symptoms (SS) in the Taiwanese diabetes patients.

**Methods:**

A total of 1,400 diabetes patients received a health examination together with a structured questionnaire interview for three categories of abnormal sensation of numbness or tingling pain, electric shock, and skin thickness sensation on seven anatomical sites on upper limbs and six sites on lower limbs. Prevalence of SS was defined using nine different criteria, with the least stringent criterion of “any positive symptom on at least 1 site” and the most stringent criterion of “any positive symptom on at least bilateral and symmetrical 2 sites involving the lower limb.” Logistic regression was used to estimate the odds ratios and their 95% confidence interval for SS by the different definitions. Fasting plasma glucose and hemoglobin A_1c_ were entered in separate models to avoid hypercollinearity.

**Results:**

The prevalence of SS was 14.4 and 54.0% when using the most stringent and least stringent criterion, respectively. Women consistently had a significantly higher prevalence than men did. Among the three categories of symptoms, numbness or tingling pain was the most common, and fingers and toes were the most commonly involved anatomical sites. For any symptoms, 37.1% of the patients had any symptoms on the upper limbs and 41.7% had any symptoms on the lower limbs. Female sex, diabetes duration, hemoglobin A_1c_, and hypertension were associated with SS in all models.

**Conclusions:**

Taiwanese diabetes patients may have a high prevalence of SS if a structured questionnaire is used for screening. Female sex, diabetes duration, hemoglobin A_1c_, and hypertension are associated with SS.

## Introduction

Diabetes mellitus is a chronic non-communicable disease characterized by various vascular complications involving the arterial system (macrovascular complications) and the capillaries (microvascular complications). “Diabetes triopathy” has been used to refer to the microvascular complications including retinopathy, nephropathy, and neuropathy (1). Diabetic polyneuropathy (DPN) may involve the sensory, motor, and/or autonomic nervous system. Sensory neuropathy can be divided clinically into painful and non-painful subtypes (2, 3). Painful diabetic peripheral neuropathy (pDPN) may impair the patients’ mood, daily function, sleep, and quality of life; and its humanistic and economic burdens are immense (4, 5).

The prevalence of pDPN varies from 6 to 34% in different reports by using different diagnostic criteria in Europe (4). A recent phone/internet survey conducted in the USA showed an ethnic difference in the prevalence of pDPN: 49% in Hispanics, 65% in African-Americans, and 87% in Caucasians (*P* < 0.05) (6).

Epidemiological studies on DPN are rare in Asian populations. In Taipei City of Taiwan, among 217 diabetes patients derived from an epidemiological survey in 1978, 44 patients (20.3%) were diagnosed as having DPN which was defined as a motor nerve conduction velocity below the normal mean minus 2 standard deviations (1). While comparing patients with and without DPN in that study, only diabetes duration, fasting glucose level, insulin use, and blood urea nitrogen (but not creatinine level) differed significantly between the two groups (1). A nationwide hospital-based study conducted in Korea suggested a prevalence of 14.4% for pDPN in patients with type 2 diabetes mellitus (7). This Korean study identified age, female sex, glycemic control, hypertension, and previous cardiovascular events as risk factors of pDPN (7). In Myanmar, a study conducted in 975 diabetes patients who attended the outpatient clinics of four hospitals reported a prevalence rate of 33.7% for DPN and 59.5% for pDPN (8). The investigators found an association between DPN and older age, longer diabetes duration, and smoking (8). In a cross-sectional study that aimed at investigating the prevalence of diabetic retinopathy in 1,008 diabetes patients enrolled from a hospital in Shijiazhuang, Hebei, China, the investigators reported a prevalence of 11% for DPN among the patients and found a close association between DPN and diabetic retinopathy (9). Another recently published cross-sectional study that enrolled patients from 17 primary care clinics across Japan showed a prevalence of 27.7% for DPN among 9,914 patients surveyed (10). Among the patients with DPN, 61.5% had DPN-related sensory symptoms/signs, which were significantly associated with female sex, smoking, and alcohol drinking (10).

Under-diagnosis is very common for pDPN in clinical practice (11). A USA study showed that while 83% of diabetes patients might report symptoms of pDNP at study, only 41% of them had ever been diagnosed as having DPN by healthcare practitioners (11). Another Japanese study showed that although 22.1% of the 298 studied patients might have pDPN, only 36.4% of them were recognized by the physicians (12). A recent multinational survey conducted in five countries in South-East Asia indicated that there might be significant gaps between physicians and patients in the perception of pDPN (13). The investigators advocated physician-patient dialogue to maximize patient outcomes (13).

Because reports on diabetic sensory neuropathy are rare and its true prevalence requires careful and systematic evaluation, the present study aimed at investigating the prevalence and risk factors of sensory symptoms (SS) in a representative cohort of diabetes patients derived from an epidemiological study in Taiwan.

## Materials and Methods

### Study Subjects

The study was approved by an ethics committee of the Department of Health of Taiwan (DOH89-TD-1035) and the subjects participated in the study voluntarily. The participants were informed of the purpose of the study and the funding of the study by the Department of Health of Taiwan. They were able to opt out of the study according to their free will. At the time of questionnaire interview and blood sampling, signed inform consent was not required according to local regulations. More than 96% of the Taiwanese population is covered by a universal and compulsory National Health Insurance at the time of the study. A total of 256,036 diabetes patients using this health insurance were assembled from 1995 to 1998 to investigate a series of epidemiologic issues (14). Baseline data on the onset symptoms and confirmation of diabetes diagnosis were collected by a questionnaire from 93,484 patients (15). At random, 4,164 patients living in the Northern Region of Taiwan from the main cluster of 93,484 patients were selected and invited to participate in a health examination. From March 1998 to September 2002, a total of 1,441 patients participated in the health examination. No significant differences in age or sex were noted among the main national sample and those who participated in the health examination (16, 17).

### Questionnaire Interview for Sensory Symptoms

For those who participated in the health examination, a structured questionnaire (**Supplementary File**) was interviewed by a well-trained interviewer for the symptoms of three categories of sensory abnormalities: 1) numbness or tingling pain; 2) electric shock; and 3) skin thickness. Seven sites on each upper limb (i.e., fingertip, other parts of the finger, palm, dorsum of hand, wrist, lower arm, and upper arm) and six sites on each lower limb (toe tip, other parts of the toe, plantar surface of foot, dorsum of foot, lower leg, and thigh) were recorded for the respective symptoms.

### Measurements of Covariates

Age, sex, diabetes duration, fasting plasma glucose/hemoglobin A_1c_ (A1C), smoking, obesity, hypertension, dyslipidemia, proteinuria, and use of insulin were treated as covariates.

Diabetes duration was defined as the time period in years between the time of receiving health examination and the time when diabetes was diagnosed. Patients who smoked one or more cigarettes per day were defined as smokers.

Anthropometric factors including body height, body weight, and waist circumference were measured as described in detail previously (18, 19). Body mass index was calculated as body weight in kg divided by the square of body height in meters. Obesity was defined as a body mass index ≥25 kg/m^2^ (20), and/or a waist circumference ≥90 cm for men or ≥80 cm for women.

A mercury sphygmomanometer was used to measure blood pressure on the right arm after 20 min rest in a sitting position. Definition of hypertension was based on one of the following three criteria: 1) being under treatment with antihypertensive drugs; 2) having systolic blood pressure ≥140 mmHg; or 3) having diastolic blood pressure ≥90 mmHg.

Subjects were instructed to avoid any vigorous physical activities one day before attending the health examination, to prevent any undue influence on the urinary excretion of albumin. In the early morning of the date of health examination, urine and blood samples were collected after fasting for a minimum duration of 12 h. First voided mid-stream urine was collected and then venous blood sample was taken. Urinary albumin concentration was measured by a particle-enhanced turbidimetric immunoassay (Biolatex^®^, Logroño, Spain) (21, 22) and urinary creatinine concentration was measured after dilution (×10) on an automated chemistry analyzer (Cobas Mira S, Roche Diagnostica, Basel, Switzerland) with reagents obtained from Randox Laboratories Ltd. (Antrim, UK). Proteinuria was defined by an albumin-to-creatinine ratio ≥300 μg/mg. Fasting plasma glucose and serum lipid profiles were measured by an automatic biochemistry analyzer (Cobas Mira S, Roche Diagnostica, Basel, Switzerland) with reagents obtained from Randox Laboratories Ltd. (Antrim, UK). A1C was measured by means of boronate affinity chromatography with reagents obtained from the Primus Corporation (Primus CLC385, Kansas City, MO, USA). Dyslipidemia was defined as a triglyceride level ≥1.7 mmol/L and/or low-density lipoprotein cholesterol ≥2.59 mmol/L and/or high-density lipoprotein cholesterol <0.9 mmol/L for men or <1.0 mmol/L for women, and/or those undergoing treatment for lipid disorder.

### Statistical Analyses

Analyses were conducted using SAS statistical software, version 9.4 (SAS Institute, Cary, NC, USA). *P*-value <0.05 was considered statistically significant.

The distributions of the three categories of sensory abnormalities (i.e., numbness or tingling pain, electric shock, and skin thickness) and any symptom by sites on upper and lower limbs were first tabulated. SS was defined by nine different criteria with positive symptom: 1) any positive symptom on at least one site; 2) any positive symptom on at least one site involving the lower limb; 3) any positive symptom on at least two sites with at least one involving the lower limb; 4) any positive symptom on at least three sites with at least one involving the lower limb; 5) any positive symptom on at least four sites with at least one involving the lower limb; 6) any positive symptom on at least two sites with at least two involving the lower limb; 7) any positive symptom on at least three sites with at least two involving the lower limb; 8) any positive symptom on at least four sites with at least two involving the lower limb; and 9) any positive symptom on at least bilateral and symmetrical two sites involving the lower limb.

The prevalence of SS according to the different criteria was then calculated for all patients and for men and women, respectively. Chi square test was used to compare the difference of SS prevalence between men and women.

To identify potential risk factors of SS, logistic regression models were created to estimate the odds ratios and their 95% confidence intervals. SS defined by different criteria was treated as a dependent variable, and all covariates including age, sex, diabetes duration, fasting plasma glucose/A1C, smoking, obesity, hypertension, dyslipidemia, proteinuria, and use of insulin were treated as independent variables. Because fasting plasma glucose and A1C were highly correlated, separate models were created for these two covariates.

## Results

A total of 1,400 patients received questionnaire interview for the sensory symptoms. Among them, 1,395 patients received health examination and had complete data of the measured covariates.

**Table 1** shows the distribution of the three categories of sensory abnormalities by sites on the upper limbs and lower limbs, respectively. The most common complaint was numbness or tingling pain, which involved mainly the fingers or toes. For any symptoms, 37.1% of the patients had any symptoms on the upper limbs and 41.7% had any symptoms on the lower limbs.

**Table 1:** Distribution of positive sensory symptoms by sites on upper and lower limbs in 1,400 diabetes patients.

Limb/Site	Numbness or tingling pain				Electric shock				Skin thickness				Any symptoms					
	No		Yes		No		Yes		No		Yes		No			Yes		
	n	%	n	%	n	%	n	%	n	%	n	%	n		%	n	%	
**Upper limb**																		
Finger tip	1,012	72.29	388	27.7	1,323	94.50	77	5.5	1,352	96.57	48	3.4	979		69.93	421	30.1	
Other parts of finger	1,016	72.57	384	27.4	1,336	95.43	64	4.6	1,353	96.64	47	3.4	987		70.50	413	29.5	
Palm	1,177	84.07	223	15.9	1,360	97.14	40	2.9	1,366	97.57	34	2.4	1,146		81.86	254	18.1	
Dorsum of hand	1,307	93.36	93	6.6	1,377	98.36	23	1.6	1,388	99.14	12	0.9	1,287		91.93	113	8.1	
Wrist	1,342	95.86	58	4.1	1,380	98.57	20	1.4	1,386	99.00	14	1.0	1,320		94.29	80	5.7	
Lower arm	1,361	97.21	39	2.8	1,383	98.79	17	1.2	1,389	99.21	11	0.8	1,345		96.07	55	3.9	
Upper arm	1,350	96.43	50	3.6	1,378	98.43	22	1.6	1,386	99.00	14	1.0	1,333		95.21	67	4.8	
Any site	916	65.43	484	34.6	1,305	93.21	95	6.8	1,331	95.07	69	4.9	881		62.93	519	37.1	
**Lower limb**																		
Toe tip	1,049	74.93	351	25.1	1,331	95.07	69	4.9	1,349	96.36	51	3.6	1,011		72.21	389	27.8	
Other parts of toe	1,027	73.36	373	26.6	1,334	95.29	66	4.7	1,318	94.14	82	5.9	972		69.43	428	30.6	
Plantar surface of foot	1,110	79.29	290	20.7	1,342	95.86	58	4.1	1,303	93.07	97	6.9	1,036		74.00	364	26.0	
Dorsum of foot	1,264	90.29	136	9.7	1,361	97.21	39	2.8	1,371	97.93	29	2.1	1,231		87.93	169	12.1	
Lower leg	1,260	90.00	140	10.0	1,356	96.86	44	3.1	1,380	98.57	20	1.4	1,227		87.64	173	12.4	
Thigh	1,336	95.43	64	4.6	1,371	97.93	29	2.1	1,384	98.86	16	1.1	1,314		93.86	86	6.1	
Any site	903	64.50	497	35.5	1,286	91.86	114	8.1	1,255	89.64	145	10.4	816		58.29	584	41.7	

**Table 2** shows the prevalence of SS by using the nine different definitions. If any positive symptom on any one site was defined as SS, then 54.0% of the patients would have SS. If the most stringent definition was applied (i.e., any positive symptom on at least bilateral and symmetrical two sites involving the lower limb), then 14.4% of the patients would have SS. When comparing the prevalence between men and women, it is evident that a significantly higher prevalence was observed in women disregarding the definitions used.

**Table 2:** Prevalence rates of sensory symptoms by using different definitions in different sexes.

Definition	All patients		Men		Women		P-value*
	n/N	PR (%)	n/N	PR (%)	n/N	PR (%)	
Any positive symptom on at least 1 site	756/1,400	54.0	339/672	50.5	417/728	57.3	0.0104
Any positive symptom on at least 1 site involving the lower limb	584/1,400	41.7	258/672	38.4	326/728	44.8	0.0155
Any positive symptom on at least 2 sites with at least one involving the lower limb	498/1,400	35.6	221/672	32.9	277/728	38.1	0.0438
Any positive symptom on at least 3 sites with at least one involving the lower limb	422/1,400	30.1	184/672	27.4	238/728	32.7	0.0305
Any positive symptom on at least 4 sites with at least one involving the lower limb	346/1,400	24.7	146/672	21.7	200/728	27.5	0.0128
Any positive symptom on at least 2 sites with at least two involving the lower limb	447/1,400	31.9	193/672	28.7	254/728	34.9	0.0134
Any positive symptom on at least 3 sites with at least two involving the lower limb	390/1,400	27.9	165/672	24.6	225/728	30.9	0.0081
Any positive symptom on at least 4 sites with at least two involving the lower limb	333/1,400	23.8	139/672	20.7	194/728	26.7	0.0088
Any positive symptom on at least bilateral and symmetrical 2 sites involving the lower limb	202/1,400	14.4	82/672	12.2	120/728	16.5	0.0228

n, cases with symptoms; N, cases interviewed; PR, prevalence rate.

*Chi-square test comparing the prevalence in men and women.

**Tables 3** and **4** show the odds ratios and their 95% confidence intervals in models using the nine different definitions of SS. **Table 3** shows the models created with fasting plasma glucose and **Table 4** shows the models created with A1C. Female sex, diabetes duration, A1C, and hypertension were the four variables that were significantly associated with SS in all models. Fasting plasma glucose was not as good as A1C in the association with SS. Smoking, obesity, and dyslipidemia were not associated with SS in all models. Except for model II in **Table 4**, age was not associated with SS. Other covariates including proteinuria and use of insulin were not consistently associated with SS.

**Table 3:** Logistic regression evaluating risk factors of sensory symptoms according to different definitions (models with fasting plasma glucose).

Risk factor	Interpretation	n/N	Odds ratio	95% Confidence interval	*P*-value
**I. Any positive symptom on at least 1 site**					
Age	Every 1-yr increment	753/1,395	1.000	(0.990–1.011)	0.9431
Sex	Men *vs.* women	338/669; 415/726	0.701	(0.528–0.932)	0.0143
Diabetes duration	Every 1-yr increment	753/1,395	1.026	(1.010–1.042)	0.0011
Fasting glucose	Every 1-mg/dl increment	753/1,395	1.002	(0.999–1.003)	0.0694
Smoking	Yes *vs.* no	262/492; 491/903	1.280	(0.956–1.716)	0.0977
Obesity	Yes *vs.* no	535/966; 218/429	1.070	(0.841–1.361)	0.5811
Hypertension	Yes *vs.* no	468/821; 285/574	1.259	(1.001–1.583)	0.0495
Dyslipidemia	Yes *vs.* no	489/886; 264/509	0.824	(0.606–1.121)	0.2185
Proteinuria	Yes *vs.* no	97/148; 656/1,247	1.512	(1.048–2.181)	0.0269
Use of insulin	Yes *vs.* no	126/196; 627/1,199	1.354	(0.970–1.889)	0.0746
**II. Any positive symptom on at least 1 site involving the lower limb**					
Age	Every 1-yr increment	581/1,395	1.010	(0.999–1.021)	0.0542
Sex	Men *vs.* women	257/669; 324/726	0.687	(0.512–0.922)	0.0123
Diabetes duration	Every 1-yr increment	581/1,395	1.024	(1.008–1.039)	0.0024
Fasting glucose	Every 1-mg/dl increment	581/1,395	1.002	(0.999–1.003)	0.0723
Smoking	Yes *vs.* no	203/492; 378/903	1.319	(0.975–1.785)	0.0726
Obesity	Yes *vs.* no	406/966; 175/429	0.895	(0.700–1.145)	0.3784
Hypertension	Yes *vs.* no	371/821; 210/574	1.290	(1.021–1.631)	0.0330
Dyslipidemia	Yes *vs.* no	381/886; 200/509	0.837	(0.614–1.140)	0.2578
Proteinuria	Yes *vs.* no	83/148; 498/1,247	1.731	(1.214–2.470)	0.0025
Use of insulin	Yes *vs.* no	104/196; 477/1,199	1.478	(1.067–2.047)	0.0187
**III. Any positive symptom on at least 2 sites with at least one involving the lower limb**					
Age	Every 1-yr increment	495/1,395	1.008	(0.998–1.019)	0.1276
Sex	Men *vs.* women	220/669; 275/726	0.711	(0.525–0.962)	0.0271
Diabetes duration	Every 1-yr increment	495/1,395	1.028	(1.013–1.044)	0.0004
Fasting glucose	Every 1-mg/dl increment	495/1,395	1.002	(0.999–1.004)	0.0512
Smoking	Yes *vs.* no	174/492; 321/903	1.308	(0.958–1.787)	0.0913
Obesity	Yes *vs.* no	340/966; 155/429	0.828	(0.644–1.065)	0.1419
Hypertension	Yes *vs.* no	317/821; 178/574	1.296	(1.018–1.651)	0.0352
Dyslipidemia	Yes *vs.* no	328/886; 167/509	1.002	(0.727–1.382)	0.9893
Proteinuria	Yes *vs.* no	66/148; 429/1,247	1.355	(0.949–1.936)	0.0950
Use of insulin	Yes *vs.* no	89/196; 406/1,199	1.393	(1.004–1.934)	0.0473
**IV. Any positive symptom on at least 3 sites with at least one involving the lower limb**					
Age	Every 1-yr increment	419/1,395	1.006	(0.995–1.018)	0.2922
Sex	Men *vs.* women	183/669; 236/726	0.699	(0.509–0.961)	0.0273
Diabetes duration	Every 1-yr increment	419/1,395	1.030	(1.014–1.046)	0.0003
Fasting glucose	Every 1-mg/dl increment	419/1,395	1.002	(0.999–1.004)	0.0521
Smoking	Yes *vs.* no	145/492; 274/903	1.278	(0.921–1.773)	0.1417
Obesity	Yes *vs.* no	288/966; 131/429	0.824	(0.634–1.072)	0.1494
Hypertension	Yes *vs.* no	275/821; 144/574	1.403	(1.089–1.809)	0.0088
Dyslipidemia	Yes *vs.* no	284/886; 135/509	1.077	(0.767–1.512)	0.6681
Proteinuria	Yes *vs.* no	57/148; 362/1,247	1.331	(0.924–1.917)	0.1246
Use of insulin	Yes *vs.* no	73/196; 346/1,199	1.229	(0.876–1.725)	0.2327
**V. Any positive symptom on at least 4 sites with at least one involving the lower limb**					
Age	Every 1-yr increment	344/1,395	1.006	(0.994–1.019)	0.3085
Sex	Men *vs.* women	145/669; 199/726	0.693	(0.493–0.973)	0.0343
Diabetes duration	Every 1-yr increment	344/1,395	1.034	(1.017–1.052)	<0.0001
Fasting glucose	Every 1-mg/dl increment	344/1,395	1.002	(1.000–1.004)	0.0396
Smoking	Yes *vs.* no	114/492; 230/903	1.190	(0.837–1.691)	0.3327
Obesity	Yes *vs.* no	240/966; 104/429	0.870	(0.657–1.152)	0.3318
Hypertension	Yes *vs.* no	227/821; 117/574	1.353	(1.032–1.774)	0.0286
Dyslipidemia	Yes *vs.* no	241/886; 103/509	1.084	(0.753–1.560)	0.6648
Proteinuria	Yes *vs.* no	51/148; 293/1,247	1.480	(1.016–2.156)	0.0412
Use of insulin	Yes *vs.* no	62/196; 282/1,199	1.480	(0.848–1.724)	0.2951
**VI. Any positive symptom on at least 2 sites with at least two involving the lower limb**					
Age	Every 1-yr increment	444/1,395	1.006	(0.995–1.017)	0.2767
Sex	Men *vs.* women	192/669; 252/726	0.717	(0.525–0.980)	0.0367
Diabetes duration	Every 1-yr increment	444/1,395	1.028	(1.012–1.044)	0.0005
Fasting glucose	Every 1-mg/dl increment	444/1,395	1.003	(1.001–1.005)	0.0031
Smoking	Yes *vs.* no	150/492; 294/903	1.193	(0.865–1.646)	0.2825
Obesity	Yes *vs.* no	310/966; 134/429	0.893	(0.689–1.158)	0.2825
Hypertension	Yes *vs.* no	289/821; 155/574	1.350	(1.052–1.732)	0.0184
Dyslipidemia	Yes *vs.* no	294/886; 150/509	0.956	(0.688–1.330)	0.7914
Proteinuria	Yes *vs.* no	62/148; 382/1,247	1.416	(0.988–2.030)	0.0585
Use of insulin	Yes *vs.* no	79/196; 365/1,199	1.284	(0.920–1.794)	0.1420
**VII. Any positive symptom on at least 3 sites with at least two involving the lower limb**					
Age	Every 1-yr increment	387/1,395	1.005	(0.993–1.017)	0.3975
Sex	Men *vs.* women	164/669; 223/726	0.715	(0.517–0.989)	0.0427
Diabetes duration	Every 1-yr increment	387/1,395	1.031	(1.014–1.047)	0.0003
Fasting glucose	Every 1-mg/dl increment	387/1,395	1.003	(1.001–1.005)	0.0070
Smoking	Yes *vs.* no	127/492; 260/903	1.131	(0.808–1.583)	0.4738
Obesity	Yes *vs.* no	270/966; 117/429	0.879	(0.671–1.152)	0.3505
Hypertension	Yes *vs.* no	255/821; 132/574	1.388	(1.070–1.801)	0.0135
Dyslipidemia	Yes *vs.* no	263/886; 124/509	1.038	(0.734–1.470)	0.8315
Proteinuria	Yes *vs.* no	54/148; 333/1,247	1.357	(0.937–1.964)	0.1060
Use of insulin	Yes *vs.* no	68/196; 319/1,199	1.199	(0.849–1.693)	0.3037
**VIII. Any positive symptom on at least 4 sites with at least two involving the lower limb**					
Age	Every 1-yr increment	331/1,395	1.006	(0.993–1.018)	0.3612
Sex	Men *vs.* women	138/669; 193/726	0.692	(0.491–0.977)	0.0362
Diabetes duration	Every 1-yr increment	331/1,395	1.033	(1.016–1.051)	0.0001
Fasting glucose	Every 1-mg/dl increment	331/1,395	1.002	(1.000–1.004)	0.0188
Smoking	Yes *vs.* no	108/492; 223/903	1.154	(0.808–1.648)	0.4315
Obesity	Yes *vs.* no	231/966; 100/429	0.868	(0.652–1.153)	0.3282
Hypertension	Yes *vs.* no	218/821; 113/574	1.334	(1.014–1.756)	0.0395
Dyslipidemia	Yes *vs.* no	232/886; 99/509	1.085	(0.749–1.570)	0.6662
Proteinuria	Yes *vs.* no	50/148; 281/1,247	1.515	(1.038–2.213)	0.0315
Use of insulin	Yes *vs.* no	61/196; 270/1,199	1.250	(0.875–1.787)	0.2202
**IX. Any positive symptom on at least bilateral and symmetrical 2 sites involving the lower limb**					
Age	Every 1-yr increment	201/1,395	1.006	(0.990–1.021)	0.4602
Sex	Men *vs.* women	81/669; 120/726	0.565	(0.365–0.875)	0.0105
Diabetes duration	Every 1-yr increment	201/1,395	1.049	(1.028–1.070)	<0.0001
Fasting glucose	Every 1-mg/dl increment	201/1,395	1.002	(0.999–1.004)	0.0908
Smoking	Yes *vs.* no	69/492; 132/903	1.500	(0.959–2.347)	0.0759
Obesity	Yes *vs.* no	137/966; 64/429	0.746	(0.529–1.053)	0.0956
Hypertension	Yes *vs.* no	142/821; 59/574	1.654	(1.171–2.336)	0.0043
Dyslipidemia	Yes *vs.* no	146/886; 55/509	1.473	(0.900–2.413)	0.1236
Proteinuria	Yes *vs.* no	34/148; 167/1,247	1.582	(1.025–2.443)	0.0385
Use of insulin	Yes *vs.* no	38/196; 163/1,199	1.139	(0.744–1.741)	0.5494

n, cases with sensory symptoms; N, cases studied.

**Table 4:** Logistic regression evaluating risk factors of sensory symptoms according to different definitions (models with A1C).

Risk factor	Interpretation	n/N	Odds ratio	95% Confidence interval	*P*-value
**I. Any positive symptom on at least 1 site**					
Age	Every 1-yr increment	753/1,395	1.001	(0.991–1.012)	0.8035
Sex	Men *vs.* women	338/669; 415/726	0.702	(0.529–0.933)	0.0149
Diabetes duration	Every 1-yr increment	753/1,395	1.025	(1.010–1.041)	0.0015
A1C	Every 1% increment	753/1,395	1.095	(1.033–1.161)	0.0023
Smoking	Yes *vs.* no	262/492; 491/903	1.277	(0.952–1.712)	0.1022
Obesity	Yes *vs.* no	535/966; 218/429	1.059	(0.832–1.348)	0.6395
Hypertension	Yes *vs.* no	468/821; 285/574	1.267	(1.007–1.595)	0.0434
Dyslipidemia	Yes *vs.* no	489/886; 264/509	0.821	(0.604–1.117)	0.2090
Proteinuria	Yes *vs.* no	97/148; 656/1,247	1.483	(1.027–2.140)	0.0356
Use of insulin	Yes *vs.* no	126/196; 627/1,199	1.336	(0.957–1.866)	0.0884
**II. Any positive symptom on at least 1 site involving the lower limb**					
Age	Every 1-yr increment	581/1,395	1.011	(1.001–1.022)	0.0381
Sex	Men *vs.* women	257/669; 324/726	0.687	(0.512–0.922)	0.0125
Diabetes duration	Every 1-yr increment	581/1,395	1.023	(1.008–1.039)	0.0031
A1C	Every 1% increment	581/1,395	1.086	(1.025–1.152)	0.0055
Smoking	Yes *vs.* no	203/492; 378/903	1.318	(0.974–1.785)	0.0739
Obesity	Yes *vs.* no	406/966; 175/429	0.885	(0.692–1.133)	0.3328
Hypertension	Yes *vs.* no	371/821; 210/574	1.300	(1.029–1.644)	0.0282
Dyslipidemia	Yes *vs.* no	381/886; 200/509	0.835	(0.613–1.137)	0.2518
Proteinuria	Yes *vs.* no	83/148; 498/1,247	1.703	(1.193–2.431)	0.0034
Use of insulin	Yes *vs.* no	104/196; 477/1,199	1.463	(1.056–2.027)	0.0222
**III. Any positive symptom on at least 2 sites with at least one involving the lower limb**					
Age	Every 1-yr increment	495/1,395	1.009	(0.999–1.021)	0.0888
Sex	Men *vs.* women	220/669; 275/726	0.712	(0.525–0.964)	0.0281
Diabetes duration	Every 1-yr increment	495/1,395	1.028	(1.012–1.044)	0.0005
A1C	Every 1% increment	495/1,395	1.099	(1.035–1.167)	0.0019
Smoking	Yes *vs.* no	174/492; 321/903	1.307	(0.956–1.786)	0.0933
Obesity	Yes *vs.* no	340/966; 155/429	0.817	(0.635–1.052)	0.1172
Hypertension	Yes *vs.* no	317/821; 178/574	1.307	(1.027–1.665)	0.0298
Dyslipidemia	Yes *vs.* no	328/886; 167/509	1.000	(0.725–1.378)	0.9987
Proteinuria	Yes *vs.* no	66/148; 429/1,247	1.329	(0.930–1.900)	0.1186
Use of insulin	Yes *vs.* no	89/196; 406/1,199	1.378	(0.993–1.914)	0.0554
**IV. Any positive symptom on at least 3 sites with at least one involving the lower limb**					
Age	Every 1-yr increment	419/1,395	1.007	(0.996–1.019)	0.2268
Sex	Men *vs.* women	183/669; 236/726	0.700	(0.509–0.963)	0.0283
Diabetes duration	Every 1-yr increment	419/1,395	1.029	(1.013–1.046)	0.0004
A1C	Every 1% increment	419/1,395	1.097	(1.032–1.167)	0.0032
Smoking	Yes *vs.* no	145/492; 274/903	1.277	(0.920–1.773)	0.1434
Obesity	Yes *vs.* no	288/966; 131/429	0.813	(0.625–1.058)	0.1242
Hypertension	Yes *vs.* no	275/821; 144/574	1.416	(1.099–1.825)	0.0072
Dyslipidemia	Yes *vs.* no	284/886; 135/509	1.075	(0.766–1.510)	0.6743
Proteinuria	Yes *vs.* no	57/148; 362/1,247	1.307	(0.907–1.885)	0.1509
Use of insulin	Yes *vs.* no	73/196; 346/1,199	1.217	(0.867–1.709)	0.2567
**V. Any positive symptom on at least 4 sites with at least one involving the lower limb**					
Age	Every 1-yr increment	344/1,395	1.007	(0.995–1.020)	0.2436
Sex	Men *vs.* women	145/669; 199/726	0.694	(0.494–0.976)	0.0357
Diabetes duration	Every 1-yr increment	344/1,395	1.034	(1.017–1.051)	<0.0001
A1C	Every 1% increment	344/1,395	1.106	(1.036–1.180)	0.0026
Smoking	Yes *vs.* no	114/492; 230/903	1.189	(0.836–1.691)	0.3346
Obesity	Yes *vs.* no	240/966; 104/429	0.857	(0.647–1.136)	0.2843
Hypertension	Yes *vs.* no	227/821; 117/574	1.367	(1.042–1.792)	0.0238
Dyslipidemia	Yes *vs.* no	241/886; 103/509	1.083	(0.752–1.559)	0.6681
Proteinuria	Yes *vs.* no	51/148; 293/1,247	1.454	(0.997–2.120)	0.0518
Use of insulin	Yes *vs.* no	62/196; 282/1,199	1.197	(0.839–1.707)	0.3220
**VI. Any positive symptom on at least 2 sites with at least two involving the lower limb**					
Age	Every 1-yr increment	444/1,395	1.006	(0.995–1.018)	0.2641
Sex	Men *vs.* women	192/669; 252/726	0.713	(0.522–0.974)	0.0334
Diabetes duration	Every 1-yr increment	444/1,395	1.028	(1.012–1.044)	0.0007
A1C	Every 1% increment	444/1,395	1.106	(1.041–1.175)	0.0012
Smoking	Yes *vs.* no	150/492; 294/903	1.198	(0.868–1.653)	0.2717
Obesity	Yes *vs.* no	310/966; 134/429	0.878	(0.677–1.139)	0.3284
Hypertension	Yes *vs.* no	289/821; 155/574	1.370	(1.068–1.758)	0.0132
Dyslipidemia	Yes *vs.* no	294/886; 150/509	0.965	(0.694–1.342)	0.8330
Proteinuria	Yes *vs.* no	62/148; 382/1,247	1.400	(0.977–2.008)	0.0671
Use of insulin	Yes *vs.* no	79/196; 365/1,199	1.270	(0.909–1.774)	0.1608
**VII. Any positive symptom on at least 3 sites with at least two involving the lower limb**					
Age	Every 1-yr increment	387/1,395	1.006	(0.994–1.017)	0.3553
Sex	Men *vs.* women	164/669; 223/726	0.713	(0.515–0.986)	0.0410
Diabetes duration	Every 1-yr increment	387/1,395	1.030	(1.013–1.047)	0.0004
A1C	Every 1% increment	387/1,395	1.111	(1.043–1.183)	0.0011
Smoking	Yes *vs.* no	127/492; 260/903	1.134	(0.810–1.587)	0.4649
Obesity	Yes *vs.* no	270/966; 117/429	0.865	(0.660–1.134)	0.2929
Hypertension	Yes *vs.* no	255/821; 132/574	1.408	(1.085–1.827)	0.0100
Dyslipidemia	Yes *vs.* no	263/886; 124/509	1.044	(0.738–1.478)	0.8082
Proteinuria	Yes *vs.* no	54/148; 333/1,247	1.044	(0.923–1.937)	0.1241
Use of insulin	Yes *vs.* no	68/196; 319/1,199	1.186	(0.839–1.674)	0.3338
**VIII. Any positive symptom on at least 4 sites with at least two involving the lower limb**					
Age	Every 1-yr increment	331/1,395	1.006	(0.994–1.019)	0.3209
Sex	Men *vs.* women	138/669; 193/726	0.691	(0.490–0.975)	0.0356
Diabetes duration	Every 1-yr increment	331/1,395	1.033	(1.015–1.050)	0.0002
A1C	Every 1% increment	331/1,395	1.102	(1.032–1.178)	0.0037
Smoking	Yes *vs.* no	108/492; 223/903	1.156	(0.809–1.652)	0.4246
Obesity	Yes *vs.* no	231/966; 100/429	0.854	(0.642–1.136)	0.2776
Hypertension	Yes *vs.* no	218/821; 113/574	1.351	(1.027–1.778)	0.0316
Dyslipidemia	Yes *vs.* no	232/886; 99/509	1.089	(0.753–1.577)	0.6499
Proteinuria	Yes *vs.* no	50/148; 281/1,247	1.495	(1.023–2.185)	0.0376
Use of insulin	Yes *vs.* no	61/196; 270/1,199	1.239	(0.867–1.770)	0.2401
**IX. Any positive symptom on at least bilateral and symmetrical 2 sites involving the lower limb**					
Age	Every 1-yr increment	201/1,395	1.006	(0.991–1.022)	0.4356
Sex	Men *vs.* women	81/669; 120/726	0.565	(0.365–0.875)	0.0105
Diabetes duration	Every 1-yr increment	201/1,395	1.048	(1.028–1.069)	<0.0001
A1C	Every 1% increment	201/1,395	1.084	(1.001–1.175)	0.0474
Smoking	Yes *vs.* no	69/492; 132/903	1.504	(0.962–2.352)	0.0737
Obesity	Yes *vs.* no	137/966; 64/429	0.734	(0.520–1.036)	0.0786
Hypertension	Yes *vs.* no	142/821; 59/574	1.672	(1.185–2.360)	0.0035
Dyslipidemia	Yes *vs.* no	146/886; 55/509	1.479	(0.903–2.421)	0.1197
Proteinuria	Yes *vs.* no	34/148; 167/1,247	1.569	(1.016–2.423)	0.0423
Use of insulin	Yes *vs.* no	38/196; 163/1,199	1.134	(0.742–1.734)	0.5612

n, cases with sensory symptoms; N, cases studied.

## Discussion

This is the first population-based observational study evaluating the sensory abnormalities in Taiwanese diabetes patients. The prevalence of 14.4% by using the most stringent criterion in the present study was lower than the reported 20.3% in our early study conducted in 1978 in Taipei City by using a definition of a slowed motor nerve conduction velocity (1). However, this prevalence rate was the same as that reported in the nationwide hospital-based survey conducted in Korea (7). The identified independent risk factors of female sex and hypertension in the present study (**Tables 3** and **4**) were also observed in the Korean study (7). The present study suggested that diabetes duration was significantly associated with SS after multivariate adjustment (**Tables 3** and **4**). On the contrary, the Korean study showed that age but not diabetes duration was significantly associated with pDPN (7). Glycemic control, especially when indicated by A1C, was significantly associated with SS in the present study (**Tables 3** and **4**), but the Korean study suggested that fasting plasma glucose was better associated with pDPN than A1C (7). The higher risk associated with the use of insulin in our previous study (1) and in the present cross-sectional study in some models (**Tables 3** and **4**) might just indicate a poor glycemic control in patients who required insulin for treatment rather than a true cause-effect relationship.

Sex differences in sensory perception have long been recognized either in animals or humans (23–25). Females are more sensitive to pain than males (23–25). The real mechanisms for such a sex discrepancy in sensory perception remain to be explored, but biological, sociocultural, and psychological factors may play some roles (24, 25). Recent studies suggested a role of estrogen in the regulation of pain processing pathway with the involvement of the adaptive immune system (22, 26, 27). It is not known whether the higher prevalence of SS in women than in men was due to sex difference in the thresholds of sensory perception. A female preponderance of pDPN was also observed in the Korean study (7) and the Japanese study (10). Animal *in vitro* and *in vivo* studies suggested that estradiol may upregulate the nocisensor transient receptor potential (TRP) vanilloid 1 receptor in sensory neurons, resulting in lowered thresholds of sensation (28). Animal studies also supported that female sex may experience greater pain related to inflammation, because of the upregulation of TRP vanilloid 1, TRP ankyrin 1, and TRP melastatin 8 by prolactin; and such effects are more prominent in female than in male rats (29, 30). Studies conducted in humans also suggested sex differences in the response to mechanical pressure pain (31) and cold pressor pain (32), which might be related to the differences in sex hormone levels between men and women and during different phases of menstruation cycle in women.

DPN may develop at an early stage of hyperglycemia including the prediabetes status (33). Therefore, the nerve damages caused by high glucose levels can develop insidiously during the long period of prediabetes status. The consistency of diabetes duration (but not age) and A1C in the association with SS (**Tables 3** and **4**) suggested that the SS might be diabetes-specific and related to glycemic control.

A recent study showed an association between pDPN and nondipping in blood pressure during midnight (34). Because the circadian change in blood pressure is controlled by autonomic nerve and diabetes patients with hypertension is highly associated with non-dipping (35), the link between hypertension and SS, but not other major atherosclerotic risk factors such as smoking, obesity, and dyslipidemia (**Tables 3** and **4**), suggested a potential involvement of autonomic neuropathy.

This study has several strengths. First, it was conducted in a population-based representative cohort of diabetes patients and might be more readily used for generalization of the findings. Second, the questionnaire covered three categories of symptoms on different anatomical sites for a better description of the clinical distributions of different symptoms. Third, by using the different definitions of SS, it was possible for us to evaluate the prevalence according to different definitions (**Table 2**) and to test the consistency of findings by using different definitions (**Tables 3** and **4**).

There are some limitations. First, this study was conducted in 1990s, and therefore, it remains unknown whether the prevalence might have changed significantly in recent years. However, a long-term follow-up of the patients would allow us to conduct studies in the near future to evaluate the impact of SS on the mortality of the patients by matching the national death certificates database in Taiwan. Second, because the study was conducted at a time when the currently used tools such as LANSS (Leeds Assessment of Neuropathic Symptoms and Signs) (36) and DN4 (Douleur Neuropathique 4) (37) were not yet available, we used a questionnaire developed by ourselves. Third, we recognized that the validity and reliability of the questionnaire used in the study had not been tested. Therefore, we still need to take some effort to test the validity and reliability of this questionnaire and to examine its usefulness as a clinical tool for predicting the development of diabetes complications and mortality. The inclusion of neurological examinations and objective laboratory tests such as Achilles tendon reflex, vibration threshold and nerve conduction velocity, etc. in our future studies would be helpful for evaluating the usefulness of the questionnaire. Fourth, the cross-sectional nature of the study did not allow a direct interpretation of cause-effect relationship between the evaluated covariates and SS. Fifth, this study was not able to evaluate the prevalence of painless neuropathy, which is related to the development of diabetic foot (38). Sixth, symptom severity was not evaluated in the present study. Seventh, the abnormal sensation obtained from the interview might not be really due to DPN. Other diseases such as carpal tunnel syndrome or spinal stenosis could not be excluded because of similar sensory presentations.

In summary, the presentation of SS is very common in the Taiwanese diabetes patients. The prevalence may range from 14.4 to 54.0% by using the most stringent criterion to using the least stringent criterion. Women are more prone to report sensory abnormalities than men. SS may be diabetes-specific and related to female sex, diabetes duration, A1C, and hypertension. However, age, and other atherosclerotic risk factors such as smoking, obesity, and dyslipidemia are not associated with SS. Prospective cohort studies are required to explore the cause-effect relationship of some covariates.

## Data Availability Statement

The datasets presented in this article are not readily available because according to the Personal Information Protection Act enacted in Taiwan, individualized data cannot be released for the protection of privacy. Requests to access the datasets should be directed to ccktsh@ms6.hinet.net.

## Ethics Statement

The studies involving human participants were reviewed and approved by the Department of Health, Taiwan. Written informed consent from the participants’ legal guardian/next of kin was not required to participate in this study in accordance with the national legislation and the institutional requirements.

## Author Contributions

C-HT researched the data and wrote manuscript. C-KC designed the questionnaire and trained interviewers. J-JS designed the questionnaire and controlled the quality of the questionnaire. All authors contributed to the article and approved the submitted version.

## Funding

The study was supported by the Department of Health (DOH89-TD-1035 and DOH97-TD-D-113-97009) of Taiwan. Analyses of the data were supported partly by the Ministry of Science and Technology (MOST 107-2221-E-002-129-MY3) of Taiwan, the Yee Fong Charity Foundation, and the Pfizer Biopharmaceuticals Group, Taiwan. The funders had no role in the study design, data collection and analysis, decision to publish, or preparation of the manuscript.

## Conflict of Interest

The authors declare that the research was conducted in the absence of any commercial or financial relationships that could be construed as a potential conflict of interest.
