# Supplementary material for: Prevalence and Risk Factors of Sensory Symptoms in Diabetes Patients in Taiwan
Source: Front Endocrinol (Lausanne). 2021 Jan 8;11:580426. doi: 10.3389/fendo.2020.580426 (PMC7821276; doi:10.3389/fendo.2020.580426)
Supplement: Supplementary file 1 [file Table_1.docx]

The following questionnaire for diabetic polyneuropathy-related sensory symptoms is an English version translated from the original Chinese version used for the study.

Questionnaire for diabetic polyneuropathy-related sensory symptoms (1/3):

Numbness or tingling pain

| **Upper limb** | | | |
| --- | --- | --- | --- |
| Finger tip | - Left | - Right | - Absent |
| Other parts of the finger | - Left | - Right | - Absent |
| Palm | - Left | - Right | - Absent |
| Dorsum of hand | - Left | - Right | - Absent |
| Wrist | - Left | - Right | - Absent |
| Lower arm | - Left | - Right | - Absent |
| Upper arm | - Left | - Right | - Absent |
| **Lower limb** | | | |
| Toe tip | - Left | - Right | - Absent |
| Other parts of the toe | - Left | - Right | - Absent |
| Plantar surface of foot | - Left | - Right | - Absent |
| Dorsum of foot | - Left | - Right | - Absent |
| Lower leg | - Left | - Right | - Absent |
| Thigh | - Left | - Right | - Absent |

Questionnaire for diabetic polyneuropathy-related sensory symptoms (2/3):

Electric shock

| **Upper limb** | | | |
| --- | --- | --- | --- |
| Finger tip | - Left | - Right | - Absent |
| Other parts of the finger | - Left | - Right | - Absent |
| Palm | - Left | - Right | - Absent |
| Dorsum of hand | - Left | - Right | - Absent |
| Wrist | - Left | - Right | - Absent |
| Lower arm | - Left | - Right | - Absent |
| Upper arm | - Left | - Right | - Absent |
| **Lower limb** | | | |
| Toe tip | - Left | - Right | - Absent |
| Other parts of the toe | - Left | - Right | - Absent |
| Plantar surface of foot | - Left | - Right | - Absent |
| Dorsum of foot | - Left | - Right | - Absent |
| Lower leg | - Left | - Right | - Absent |
| Thigh | - Left | - Right | - Absent |

Questionnaire for diabetic polyneuropathy-related sensory symptoms (3/3):

Skin thickness

| **Upper limb** | | | |
| --- | --- | --- | --- |
| Finger tip | - Left | - Right | - Absent |
| Other parts of the finger | - Left | - Right | - Absent |
| Palm | - Left | - Right | - Absent |
| Dorsum of hand | - Left | - Right | - Absent |
| Wrist | - Left | - Right | - Absent |
| Lower arm | - Left | - Right | - Absent |
| Upper arm | - Left | - Right | - Absent |
| **Lower limb** | | | |
| Toe tip | - Left | - Right | - Absent |
| Other parts of the toe | - Left | - Right | - Absent |
| Plantar surface of foot | - Left | - Right | - Absent |
| Dorsum of foot | - Left | - Right | - Absent |
| Lower leg | - Left | - Right | - Absent |
| Thigh | - Left | - Right | - Absent |
